# The Role of METTL3-Mediated m^6^A Methylation in Hepatocellular Carcinoma Progression and Treatment

**DOI:** 10.3390/epigenomes10040063

**Published:** 2026-10-04

**Authors:** Bala Gamage Nalaka Hasith Maduranga, Hee Doo Yang, Su In Lee, Jung Woo Eun, Sun-Young Lim, Hyung Seok Kim, Jee-Yeong Jeong

**Affiliations:** 1 Department of Marine Bioscience and Environment, College of Ocean Science and Technology, Korea Maritime & Ocean University, Busan 49112, Republic of Korea; nalakahasith@gmail.com (B.G.N.H.M.); sylim@kmou.ac.kr (S.-Y.L.); 2 Section of Hematology, Department of Internal Medicine and Yale Cancer Center, Yale University School of Medicine, New Haven, CT 06520, USA; heedoo.yang@yale.edu; 3 Department of Biochemistry, Kosin University College of Medicine, Seo-Gu, Busan 49267, Republic of Korea; lsi8906@naver.com; 4 Department of Gastroenterology, Ajou University School of Medicine, 164 Worldcup-Ro, Yeongtong-Gu, Suwon 16499, Republic of Korea; jetaimebin@gmail.com

**Keywords:** epitranscriptomics, hepatocellular carcinoma, N^6^-methyladenosine, METTL3, tumor microenvironment

## Abstract

Hepatocellular carcinoma (HCC) is a major cause of cancer-related mortality worldwide. Emerging evidence has established N^6^-methyladenosine (m^6^A) RNA methylation as a key epitranscriptomic mechanism underlying hepatocarcinogenesis. Methyltransferase-like 3 (METTL3), the catalytic core of the m^6^A methyltransferase complex (MTC), forms a heterodimer with METTL14 and cooperates with auxiliary components of the MTC to deposit m^6^A modifications that modulate mRNA stability, splicing, nuclear export, and translation. In this review, we comprehensively summarize the current understanding of METTL3 biology in HCC, focusing on its structure, assembly within the MTC, regulation of coding and non-coding RNAs, and its roles in driving key oncogenic signaling cascades, such as JAK/STAT3, PI3K/AKT/mTOR, Wnt/β-catenin, Hippo/YAP, Notch, Hedgehog, and epithelial–mesenchymal transition. In addition, we discussed how METTL3 remodels the tumor immune microenvironment (TIME) by regulating immune responses and metabolic pathways promoting immune evasion and therapy resistance. Therapeutic strategies targeting METTL3—including small-molecule inhibitors, CRISPR-Cas9 approaches, combination immunotherapy, and tumor-selective delivery systems—are discussed together with the challenges that continue to limit clinical translation. Collectively, these findings position METTL3 as a central epitranscriptomic regulator of HCC progression; however, further mechanistic and clinical validation remains necessary to establish its utility as a therapeutic target and prognostic biomarker.

## 1. Introduction

Epitranscriptomics is the study of biochemical modifications on RNA molecules that regulate gene expression and RNA metabolic processes without altering the genetic sequence [1,2,3]. Numerous distinct RNA modifications have been identified to date, of which m^6^A is the most abundant and well-characterized modification in eukaryotic mRNA [4,5]. The m^6^A methylation functions as a dynamic and reversible epitranscriptomic regulatory mechanism, regulated by the interplay between “writers” (methyltransferases), “erasers” (demethylases), and “readers” (m^6^A-binding proteins) [6,7]. m^6^A is predominantly enriched within the 3′-untranslated regions (UTR) and near stop codons of mRNAs, where it regulates post-transcriptional gene regulation [1,5]. This modification regulates post-transcriptional processes, such as mRNA stability, alternative splicing, localization, translational efficiency, and cellular responses to oncogenic signaling [8,9,10]. Dysregulation of m^6^A homeostasis drives the initiation and progression of many cancers. Dysregulated m^6^A methylation disrupts the post-transcriptional stability and translation of oncogenic transcripts, resulting in broad transcriptomic alterations that promote cancer cell proliferation, metabolic reprogramming, immune evasion, and resistance to anticancer therapies, sustaining tumor growth [3,11,12,13]. In line with these findings, aberrant expression of m^6^A regulatory proteins is commonly observed in malignant tissues and is associated with advanced tumor stage, increased invasiveness, and poor patient survival [14].

Among the components of the m^6^A machinery, METTL3 functions as the principal catalytic writer and has attracted considerable attention in cancer research as it harbors the catalytic activity required for methyl-group transfer within the m^6^A MTC [13,15,16]. METTL3 constitutes the catalytic core of the MTC by forming a stable heterodimer with METTL14, which primarily facilitates RNA substrate recognition, whereas auxiliary proteins, including WTAP, VIRMA, RBM15/15B, ZC3H13, and HAKAI, govern complex assembly, subcellular localization, substrate selectivity, and methylation efficiency [3,17,18,19,20,21]. Using S-adenosyl-L-methionine (SAM) as the universal methyl donor, the METTL3–METTL14 complex catalyzes m^6^A deposition on select target transcripts, enabling transcript-specific epitranscriptomic regulation upon recognition by m^6^A-binding proteins [2,16,22,23].

Although METTL3 has been implicated in a broad spectrum of malignancies, its biological role in hepatocarcinogenesis is context-dependent. Experimental evidence indicates that METTL3 exerts tumor-suppressive effects during the earliest stages of malignant transformation, whereas its oncogenic function predominates during established disease, depending on the transcript repertoire regulated, cellular state, and stage of hepatic tumor progression [24]. In HCC, aberrant METTL3 expression has been associated with altered regulation of oncogenic transcripts that drive tumor cell proliferation, metabolic adaptation, invasion, metastasis, and therapeutic resistance [11,13,25]. Elevated m^6^A modification has also been associated with resistance to chemotherapeutic agents and targeted therapies, including lenvatinib [26]. In addition to these tumor-intrinsic effects, METTL3 contributes to remodeling the tumor microenvironment (TME) by regulating immune and metabolic pathways, including impaired cytotoxic CD8+ T-cell infiltration and regulation of ferroptosis [9,27]. Yang et al. demonstrated that the HBXIP-METTL3 interaction contributes to HCC progression, suggesting METTL3 as a therapeutic target [28]. Taken together, these findings identify METTL3 as a key epitranscriptomic regulator in HCC and underscore its potential as a biomarker and therapeutic target.

This review examines the molecular mechanisms through which METTL3-mediated m^6^A methylation drives HCC development and progression. Specifically, it addresses the structural organization and regulation of the m^6^A MTC, METTL3-dependent coding and non-coding RNA networks, oncogenic signaling, metabolic reprogramming, ferroptosis, and TIME, as well as current therapeutic strategies and the challenges associated with METTL3-targeted therapy.

For this review, relevant literature was identified through searches of PubMed, using combinations of the terms “METTL3,” “m^6^A,” “hepatocellular carcinoma,” and “HCC,” with emphasis on studies published up to August 2026.

## 2. Molecular Architecture and Biochemical Basis of the m^6^A Writer Complex

### 2.1. Gene Structure and Protein Organization

**Figure 1:**
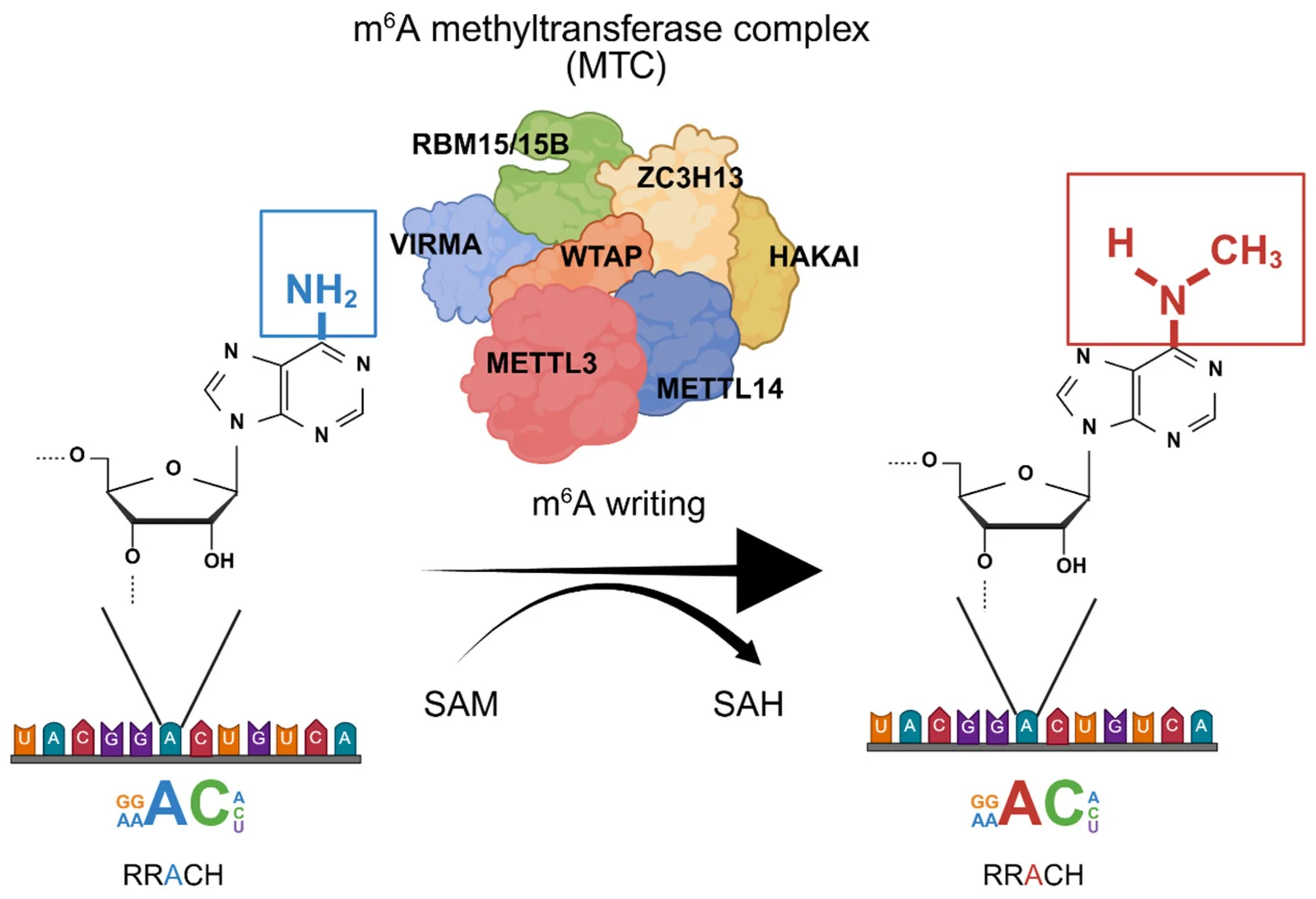
**Composition and catalytic mechanism of the m^6^A methyltransferase complex (MTC).** The MTC deposits N^6^-methyladenosine (m^6^A) modifications onto target RNA. The catalytic core consists of a METTL3–METTL14 heterodimer, stabilized by auxiliary subunits including WTAP, VIRMA, RBM15/15B, ZC3H13, and HAKAI. Within the consensus RRACH motif (R = A or G; H = A, C, or U), METTL3 transfers a methyl group from S-adenosylmethionine (SAM) to the N^6^ position of adenosine, producing N^6^-methyladenosine and S-adenosylhomocysteine (SAH). The resulting m^6^A modification influences mRNA stability, splicing, nuclear export, and translation. Created in Biorender. Hyung Seok Kim. (2026) https://BioRender.com/2sozgmh (accessed on 30 June 2026).

The METTL3 gene encodes a modular protein comprising two principal functional domains: an N-terminal CCCH-type zinc finger domain (ZFD) and a C-terminal methyltransferase domain (MTD). These domains cooperate to ensure accurate substrate recognition and efficient m^6^A deposition [29,30,31]. The ZFD binds single-stranded RNA, contributing to substrate recognition by positioning target transcripts within the catalytic center. Although the ZFD lacks direct catalytic activity, this domain confers substrate specificity and facilitates efficient m^6^A deposition [30,32]. In contrast, the MTD harbors the conserved DPPW catalytic motif and the SAM-binding pocket, which cooperatively mediate methyl-group transfer to adenosine residues within the RRACH consensus sequence (R = A/G; H = A/C/U) (Figure 1) [29].

### 2.2. Assembly of the METTL3 MTC

#### 2.2.1. METTL3–METTL14 Heterodimer

The METTL3–METTL14 heterodimer constitutes the catalytic core of the m^6^A MTC. METTL3 provides catalytic activity [16], whereas METTL14 primarily serves as a structural scaffold that enhances RNA binding, stabilizes the heterodimer, and supports accurate m^6^A deposition [21,24,29,33,34,35]. Thus, RNA substrate recognition is cooperative: METTL14 provides the major RNA-binding interface and structural support, whereas the METTL3 ZFD contributes sequence-selective engagement and positioning of the target adenosine for catalysis. Additionally, the catalytic core is stabilized and regulated by several auxiliary proteins, including WTAP, VIRMA, RBM15/15B, ZC3H13, and HAKAI, which are essential for the regulation of complex localization and substrate selection of this complex, promoting its localization to nuclear speckles and directing the complex toward specific RNA substrates [2,15]. The MTD of METTL3 adopts a Rossmann fold architecture with gate loops that coordinate SAM binding and facilitate target adenosine recognition. METTL3 functions as the catalytic subunit of the m^6^A writer complex, while structural features of the METTL protein family, including N-terminal RNA-binding domains and C-terminal VCR motifs, modulate enzymatic efficiency in response to intracellular SAM availability [36,37]. The m^6^A landscape is reversible and tightly regulated by demethylases such as FTO and ALKBH5, which demethylate target transcripts and modulate m^6^A levels [38,39].

In HCC, increased METTL3 activity shifts the epitranscriptomic balance toward m^6^A hypermethylation, thereby stabilizing oncogenic transcripts and promoting tumor progression [40,41]. Recent studies have shown that METTL3 activity can also be regulated through post-translational modification. O-GlcNAcylation of METTL3 strengthens its interaction with WTAP, thereby increasing m^6^A methyltransferase activity and promoting HCC progression via MCM10 upregulation [22].

#### 2.2.2. WTAP (Wilms’ Tumor 1-Associating Protein)

WTAP functions as a key regulatory and adaptor subunit that bridges the catalytic core of the MTC with auxiliary factors. By promoting the recruitment of the METTL3–METTL14 heterodimer to specific nuclear compartments, WTAP is required for ensuring the spatial organization and functional integrity of the complex [18,24,35,42]. WTAP is also essential for maintaining the structural integrity of the complex, as its depletion impairs the nuclear localization of METTL3 and METTL14 resulting in a global reduction in m^6^A levels [6,18].

In HCC, WTAP overexpression has been correlated with adverse clinicopathological features, including advanced tumor stage and poor survival outcomes [7,15]. Recent studies further demonstrate that WTAP drives HCC progression through selective m^6^A-dependent regulation of metabolic and ferroptosis-related transcripts. Under hypoxic conditions, WTAP-mediated m^6^A modification facilitates YTHDF2-mediated degradation of PPARGC1A mRNA, resulting in suppression of the PPARGC1A-BAMBI-ACSL5 axis. This pathway regulates Wnt/β-catenin and TGF-β/SMAD signaling, modulates reactive oxygen species (ROS) production, and regulates ferroptotic cell death, promoting HCC progression and lenvatinib resistance [27].

#### 2.2.3. VIRMA

VIRMA, also known as KIAA1429, acts as a scaffold protein that recruits the MTC to a specific RNA region and cooperates with METTL3, METTL14, and WTAP to execute m^6^A methylation [42]. VIRMA directs the complex toward 3′-UTR and regions near stop codons, enabling site-selective m^6^A deposition [2,11].

In HCC, VIRMA promotes m^6^A modification of transcripts that support tumor cell proliferation and suppress apoptosis. VIRMA overexpression is associated with enhanced tumor aggressiveness and poor clinical outcomes through post-transcriptional regulation of oncogenic pathways [43,44]. Through its interaction with CPSF5 and CPSF6, VIRMA links the m^6^A MTC to the pre-mRNA 3′-end processing machinery, coupling m^6^A deposition with alternative polyadenylation and modulation of transcript stability. These mechanisms collectively promote the expression of VIRMA-regulated oncogenic transcripts and contribute to hepatocarcinogenesis [11].

#### 2.2.4. RBM15/15B

RBM15 and RBM15B are RNA-binding proteins that bridge the association between the m^6^A MTC and target RNA substrates. By tethering the METTL3–METTL14 complex to specific motifs within nascent pre-mRNA, these proteins direct m^6^A deposition to defined RNA regions [11]. Specifically, RBM15 recognizes U-rich sequences and promotes m^6^A deposition on transcripts implicated in tumor cell proliferation and survival [4].

Recent studies have shown that RBM15 promotes METTL3-mediated m^6^A deposition on target transcripts, activating the IGF2BP1-YES1-MAPK signaling axis and driving HCC progression [33]. RBM15 modulates oncogenic signaling that sustains malignant progression, including increased tumor cell proliferation and survival.

#### 2.2.5. ZC3H13

ZC3H13 is a zinc-finger protein that coordinates the nuclear localization and structural assembly of the m^6^A MTC. By retaining this complex within the nucleus, ZC3H13 promotes interactions among its subunits, optimizing the catalytic efficiency of the MTC [11]. ZC3H13 depletion impairs the functional integrity of the MTC in HCC, resulting in reduced m^6^A levels and impaired tumor cell proliferation. ZC3H13 also promotes malignancy by controlling the nuclear export of target transcripts, restricting METTL3-mediated m^6^A deposition to the nucleus to sustain oncogenic gene expression.

In addition to its role in MTC assembly, ZC3H13 is required for m^6^A-mediated post-transcriptional regulation of transcripts that drive proliferation and malignant progression in HCC [34]. Additionally, the interaction between MTC components and m^6^A reader YTHDF1 promotes metastatic progression through stabilization of oncogenic transcripts including FOXP4 [23].

#### 2.2.6. HAKAI

HAKAI (also known as CBLL1) is an E3 ubiquitin ligase within the m^6^A MTC. It regulates the stability and turnover of the MTC, by controlling METTL3 protein levels required to maintain m^6^A homeostasis within the cell [11].

In HCC, HAKAI-mediated ubiquitination is a key regulatory mechanism; its dysregulation leads to aberrant METTL3 upregulation, which serves as an independent predictor of poor prognosis [36]. Thus, HAKAI acts as a regulatory component of the m^6^A MTC by controlling the abundance and activity of the methyltransferase complex rather than directly catalyzing m^6^A methylation.

### 2.3. Catalytic Mechanism of m^6^A

m^6^A deposition is governed by the coordinated activity of the MTC, which catalyzes methyl-group transfer to select adenosine residues within target transcripts [4]. This process occurs primarily in the nucleus and is closely coupled with transcription, enabling m^6^A deposition on nascent transcripts prior to RNA maturation. METTL3 preferentially methylates adenosine within the conserved RRACH consensus sequence [18,34,45]. Methyl-group transfer is mediated by SAM, which binds the catalytic pocket of METTL3 and donates a methyl group to the N^6^ position of the target adenosine. This reaction generates S-adenosyl-L-homocysteine (SAH) as a by-product [21,29]. Once deposited, m^6^A marks are recognized by reader proteins that regulate RNA stability, translation, and splicing, shaping transcript fate and gene expression [11].

## 3. METTL3-Mediated Molecular Mechanisms in HCC

Beyond its effects on protein-coding transcripts, METTL3 regulates multiple classes of coding and non-coding RNAs through m^6^A-dependent post-transcriptional mechanisms. These effects encompass mRNA stability and translation, microRNA (miRNA) biogenesis, long non-coding RNA (lncRNA) function, and circular RNA (circRNA) biogenesis, collectively broadening the post-transcriptional regulatory networks that drive HCC progression.

### 3.1. Regulation of Coding and Non-Coding RNAs

#### 3.1.1. Regulation of mRNA Translation and Stability

Following m^6^A deposition, these modifications serve as post-transcriptional regulators of gene expression, modulating mRNA stability, and translational efficiency. The functional consequence of m^6^A is primarily determined by the recruitment of “reader” proteins, including members of the YTHDF family and IGF2BP3, which recognize m^6^A-modified sites to either promote transcript degradation or enhance transcript stability [17,46,47]. Whereas YTHDF proteins promote transcript decay, IGF2BP3 recognizes m^6^A-modified transcripts, stabilizing them, and enhancing their translation [17].

In HCC, this regulatory axis is commonly dysregulated, resulting in selective stabilization of oncogenic transcripts that promote tumor progression [41]. LIMA1 represents one such example whereby METTL3-mediated m^6^A modification stabilizes its mRNA, activating the Wnt/β-catenin and Hippo signaling pathways in HCC [10]. This mechanism similarly affects other drivers of HCC, such as FEN1 and EGFR, in which METTL3-driven transcript stabilization promotes aggressive proliferation and resistance to lenvatinib [13,26,48]. Collectively, these findings suggest that altered transcript stability via m^6^A modification is a key mechanism sustaining the malignant phenotype and driving chemoresistance in HCC cells [41].

**Figure 2:**
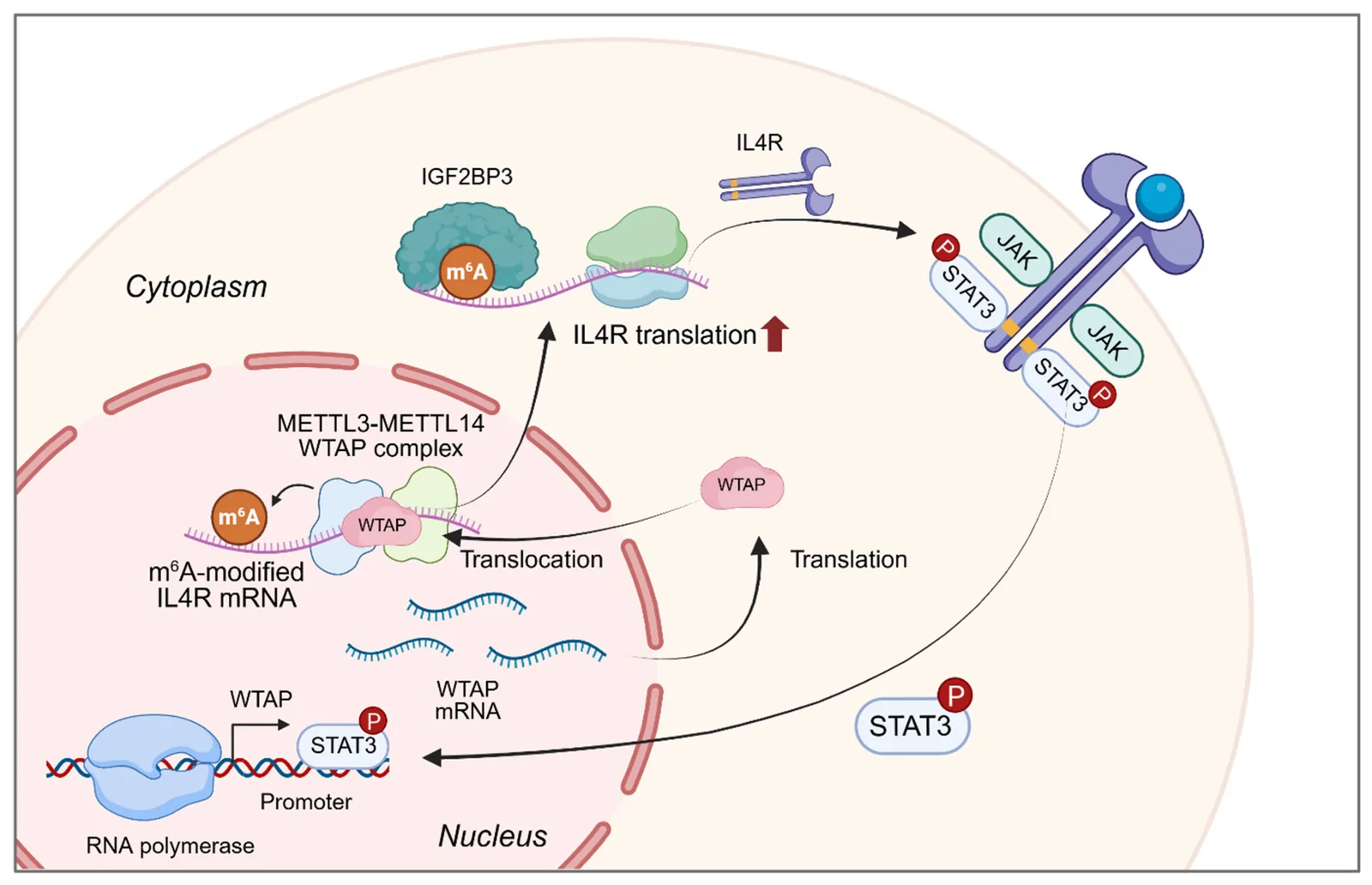
**METTL3-mediated m^6^A modification of IL4R mRNA activates JAK/STAT3 signaling.** In the nucleus, phosphorylated STAT3 binds to the WTAP promoter, driving WTAP transcription. The transcribed WTAP mRNA is then exported to the cytoplasm for translation, and the newly synthesized WTAP protein translocates back into the nucleus. Therefore, it is incorporated into the METTL3–METTL14–WTAP complex, which deposits m^6^A modifications onto IL4R mRNA. In the cytoplasm, the m^6^A reader IGF2BP3 recognizes and binds m^6^A-modified IL4R mRNA, enhancing its translation. The red arrow indicates increased IL4R translation. Elevated IL4R protein levels at the cell surface promote JAK-mediated phosphorylation of STAT3, generating a positive feedback loop that sustains STAT3 activation. Created in Biorender. Hyung Seok Kim. (2026) https://BioRender.com/ael1oas (accessed on 30 June 2026).

Beyond its role in RNA stability and alternative splicing, m^6^A modification regulates translational efficiency by promoting the association of methylated transcripts with the translation machinery [17,46]. Reader proteins, including the YTHDF family members and IGF2BP3, bind m^6^A-modified transcripts, facilitate ribosome loading, and augment protein output from target transcripts [17,46,49]. In HCC, METTL3-mediated translational control sustains the expression of proteins implicated in tumor progression and therapeutic resistance. METTL3 enhances STAT3 translation, activating the JAK/STAT signaling pathway, while STAT3-driven WTAP upregulation may further promote METTL3 localization and MTC assembly (Figure 2) [15,17]. METTL3 also increases EGFR translation, driving lenvatinib resistance in HCC cells [26,48]. These findings demonstrate that dysregulated translational control represents a key mechanism by which METTL3 drives HCC progression and modulates therapeutic response.

#### 3.1.2. Regulation of miRNAs

**Figure 3:**
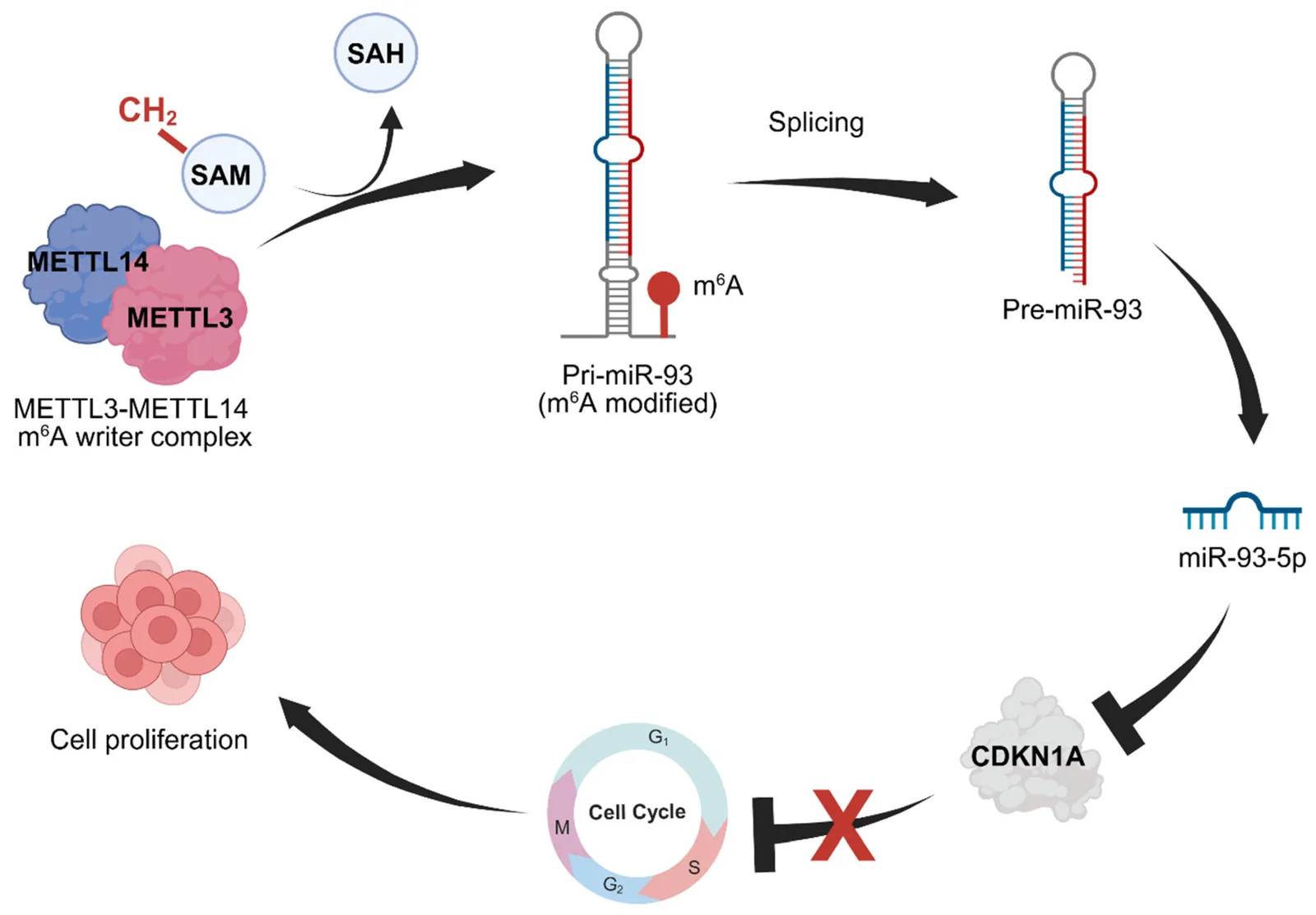
**METTL3-mediated m^6^A modification of pri-miR-93 facilitates miR-93-5p biogenesis and drives cell proliferation.** The METTL3–METTL14 m^6^A writer complex transfers a methyl group from S-adenosylmethionine (SAM) to pri-miR-93, generating S-adenosylhomocysteine (SAH) as a byproduct. The resulting m^6^A-modified pri-miR-93 undergoes processing to produce pre-miR-93, which is subsequently processed into the mature miR-93-5p. miR-93-5p suppresses CDKN1A expression, relieving its inhibitory effect on cell cycle progression (indicated by the red cross) and ultimately promoting cell proliferation. Created in Biorender. Hyung Seok Kim. (2026) https://BioRender.com/g4e7ezx (accessed on 30 June 2026).

METTL3 regulates miRNA biogenesis through m^6^A-dependent processing of primary miRNAs (pri-miRNAs). m^6^A modification facilitates the recognition and processing of pri-miRNAs by the microprocessor complex, promoting the production of mature miRNAs [50,51]. In HCC, a recent study demonstrated that METTL3-mediated m^6^A modification of pri-miR-93 enhances its maturation into miR-93-5p, suppressing CDKN1A and promoting cell proliferation, cell-cycle progression, and resistance to apoptosis (Figure 3) [46]. A similar mechanism has been reported for miR-589-5p in liver cancer. METTL3 promotes miR-589-5p maturation through m^6^A modification of pri-miR-589, as METTL3 silencing increased pri-miR-589 levels while reducing mature miR-589-5p expression. Mutation of the identified m^6^A motif in pri-miR-589 further impaired miR-589-5p maturation, confirming the dependence of this process on m^6^A modification [52]. The METTL3/miR-589-5p axis also promoted liver cancer cell viability, migration, invasion, and xenograft tumor growth [52]. METTL3 can also affect miRNA activity indirectly through m^6^A modification of circRNAs. In HBV-associated HCC, METTL3 promoted the m^6^A-dependent biogenesis of circ-ARL3, which sponged miR-1305 and contributed to tumor progression [53]. METTL3 also regulated the m^6^A modification of circ-CCT3, which promoted HCC progression by sponging miR-378a-3p [54]. Together, these findings indicate that METTL3 regulates miRNA-mediated pathways in HCC through both direct control of miRNA maturation and indirect modulation of circRNA–miRNA interactions. However, evidence for METTL3-mediated regulation of miRNA pathways in HCC remains limited, while circRNA–miRNA interactions represent a potential direction for future investigation.

#### 3.1.3. Regulation of Long Non-Coding RNAs (lncRNAs)

METTL3-mediated m^6^A modification regulates several oncogenic lncRNAs, including GBAP1, LINC00958, lnc-CTHCC, and MAPKAPK5-AS1 (MAAS), all of which promote HCC progression [55,56,57,58]. Among these, lncRNA GBAP1 is markedly overexpressed in HCC tissues [55]. METTL3 enhances GBAP1 expression and stability through m^6^A modification, promoting HCC cell proliferation, migration, and invasion via the miR-22-3p/BMPR1A/SMAD signaling pathway [55]. Similarly, METTL3 increases the stability of LINC00958 through m^6^A methylation, promoting lipogenesis through the miR-3619-5p/HDGF axis and enhancing HCC cell proliferation, migration, and invasion [58]. In addition, METTL3-mediated m^6^A modification of lnc-CTHCC is recognized by IGF2BP1 and IGF2BP3, which stabilize lnc-CTHCC and promote HCC growth and metastasis through the lnc-CTHCC/hnRNPK/YAP1 axis [57]. MAAS is another oncogenic lncRNA that is highly expressed in HCC tissues, and its elevated expression correlates with poor patient survival [56]. Furthermore, hepatitis B e antigen secreted by HCC cells promotes METTL3-mediated m^6^A modification, increasing MAAS expression in M2 macrophages. MAAS is subsequently transferred to HCC cells via M2 macrophage-derived exosomes, where it activates the MYC proto-oncogene to promote HCC cell proliferation [56].

#### 3.1.4. Regulation of circRNAs

**Figure 4:**
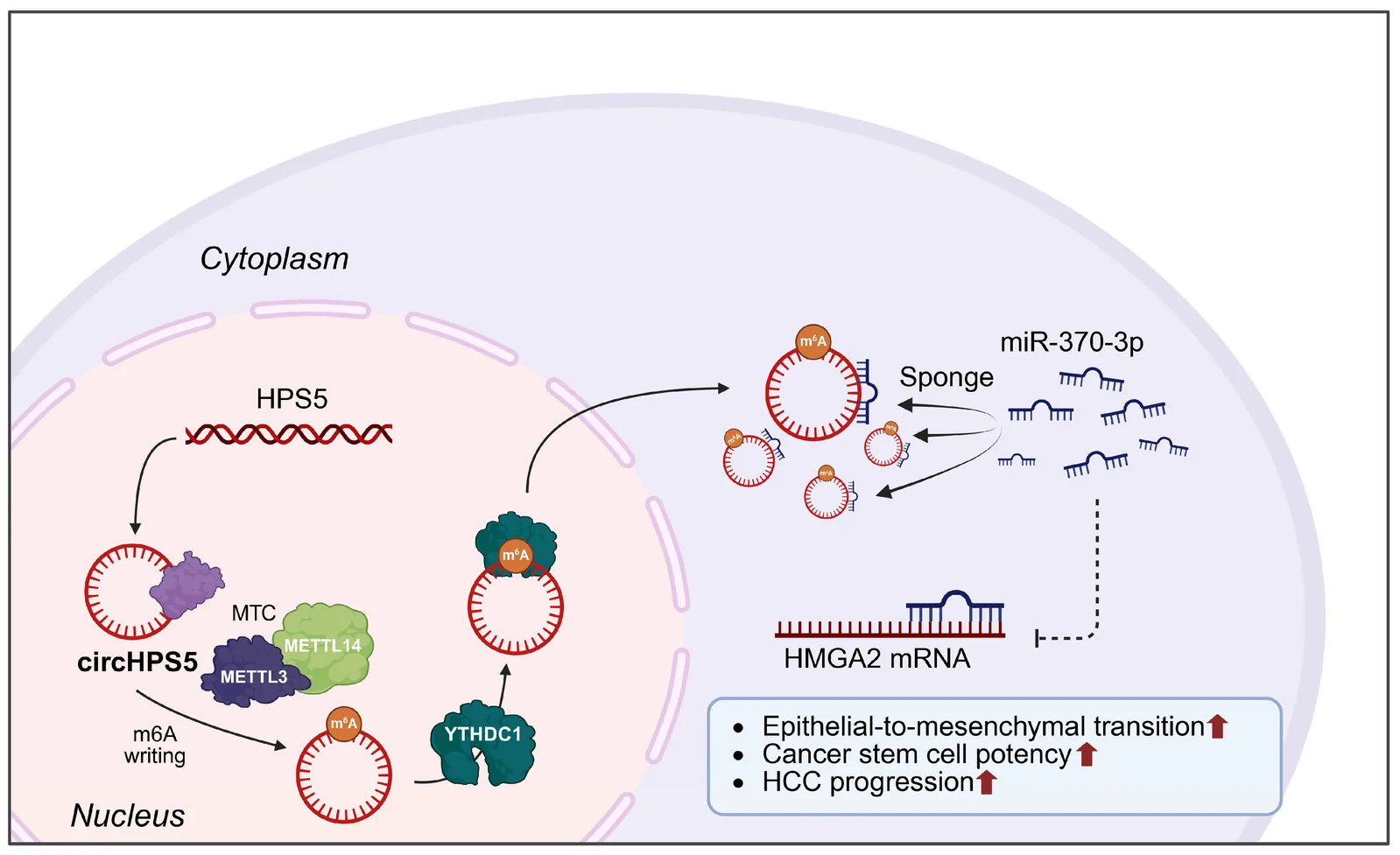
**METTL3-mediated m^6^A modification of circHPS5 drives EMT, cancer stem cell potency, and HCC progression.** In the nucleus, HPS5 pre-mRNA undergoes back-splicing to generate circHPS5, which is subsequently m^6^A-modified by the METTL3-METTL14 complex. The m^6^A reader YTHDC1 recognizes m^6^A-modified circHPS5 and facilitates its export to the cytoplasm. In the cytoplasm, circHPS5 functions as a sponge for miR-370-3p, sequestering it and relieving its suppressive effect on HMGA2 mRNA. Elevated HMGA2 expression promotes epithelial-to-mesenchymal transition (EMT), enhances cancer stem cell potency, and drives HCC progression. Red arrows indicate increased activity or upregulation of the associated biological processes or cellular characteristics. Created in Biorender. Hyung Seok Kim. (2026) https://BioRender.com/77ksdgh (accessed on 30 June 2026).

In HCC, some circRNAs function as oncogenic circRNAs, with high circRNA expression correlating with poor patient prognosis in HCC patients [53,54,59,60]. METTL3 regulates circRNA biogenesis through m^6^A-dependent modulation of back-splicing and promotes HCC progression by enhancing the expression and function of oncogenic circRNAs [59,61,62]. METTL3 increases the m^6^A modification of circ-CCT3, promoting HCC cell proliferation, migration, and invasion through the circ-CCT3/miR-378a-3p/FLT1 axis, representing a potential therapeutic target in HCC [54]. Similarly, HBx (hepatitis B virus X protein)-induced upregulation of METTL3 increases the m^6^A modification of circ-ARL3, recognized by the m^6^A reader YTHDC1, promoting its biogenesis, stability, and expression, disrupting the circ-ARL3/miR-1305 axis and facilitating HCC progression [53]. Likewise, METTL3-mediated m^6^A modification promotes the formation and accumulation of circHPS5, which is recognized by YTHDC1 facilitating its cytoplasmic export and activation of the circHPS5/miR-370/HMGA2 axis, promoting epithelial–mesenchymal transition, cancer stem-like properties, and HCC progression (Figure 4) [61]. Collectively, these findings identify METTL3-mediated m^6^A modification as a key regulator of oncogenic circRNA biogenesis in HCC.

### 3.2. METTL3-Mediated Oncogenic Signaling Pathways in HCC

#### 3.2.1. JAK/STAT3 Feedback Signaling and Metastasis

A positive feedback loop between METTL3 and STAT3 has been identified in HCC [15,17]. METTL3-mediated m^6^A modification of STAT3 mRNA enhances its translation via YTHDF1-dependent recruitment of the translational initiation machinery, increasing STAT3 protein abundance [17]. STAT3 is a transcription factor that promotes HCC progression by regulating cellular proliferation, survival, angiogenesis, and metastatic dissemination [15]. In turn, STAT3 transcriptionally upregulates WTAP, a core component of the MTC, facilitating METTL3 nuclear translocation and enhancing overall m^6^A methyltransferase activity [17]. This METTL3–STAT3 positive feedback loop amplifies oncogenic signaling and drives HCC metastasis in both in vitro and in vivo models [15,17].

#### 3.2.2. EGFR/PI3K/AKT/mTOR

METTL3 enhances EGFR/PI3K/AKT/mTOR signaling through m^6^A-mediated post-transcriptional regulation [8,33]. The EGFR/PI3K/AKT/mTOR signaling axis drives oncogenic phenotypes including uncontrolled proliferation, anabolic metabolism, survival, migration, and resistance to apoptosis [33,63,64]. It also shapes the TME regulation by promoting angiogenesis and inflammatory cell recruitment. METTL3 marks PTEN and TSC2 mRNAs for YTHDF2-mediated degradation, relieving two negative regulators of the PI3K/AKT/mTOR axis and driving sustained pathway activation [8,33]. METTL3 also promotes metabolic reprogramming to support malignant cell survival. METTL3-mediated m^6^A modification targets the 3′-UTR of TRIM21 mRNA, promoting its YTHDF2-dependent destabilization. This event prevents TRIM21-mediated ubiquitination and subsequent proteasomal degradation of G6PD, indicating that METTL3 stabilizes G6PD by impairing TRIM21-mediated proteasomal degradation [8,65]. G6PD stabilization significantly elevates the pentose phosphate pathway flux, generating the necessary NADPH and nucleotide precursors to facilitate tumor growth and confer resistance to platinum-based chemotherapies, such as oxaliplatin, as well as tyrosine kinase inhibitors like lenvatinib [8,48]. METTL3-mediated m^6^A modification of transcripts encoding serine synthesis pathway (SSP) enzymes, through an IGF2BP3-dependent stabilization mechanism, promotes activation of this metabolic axis in sorafenib-resistant HCC cells [66,67]. Activation of the m^6^A-SSP axis increases intracellular antioxidant capacity, reduces ROS levels, and promotes tumor cell survival under sorafenib-induced stress, contributing to METTL3-associated therapeutic resistance [67].

#### 3.2.3. Wnt/β-Catenin

**Figure 5:**
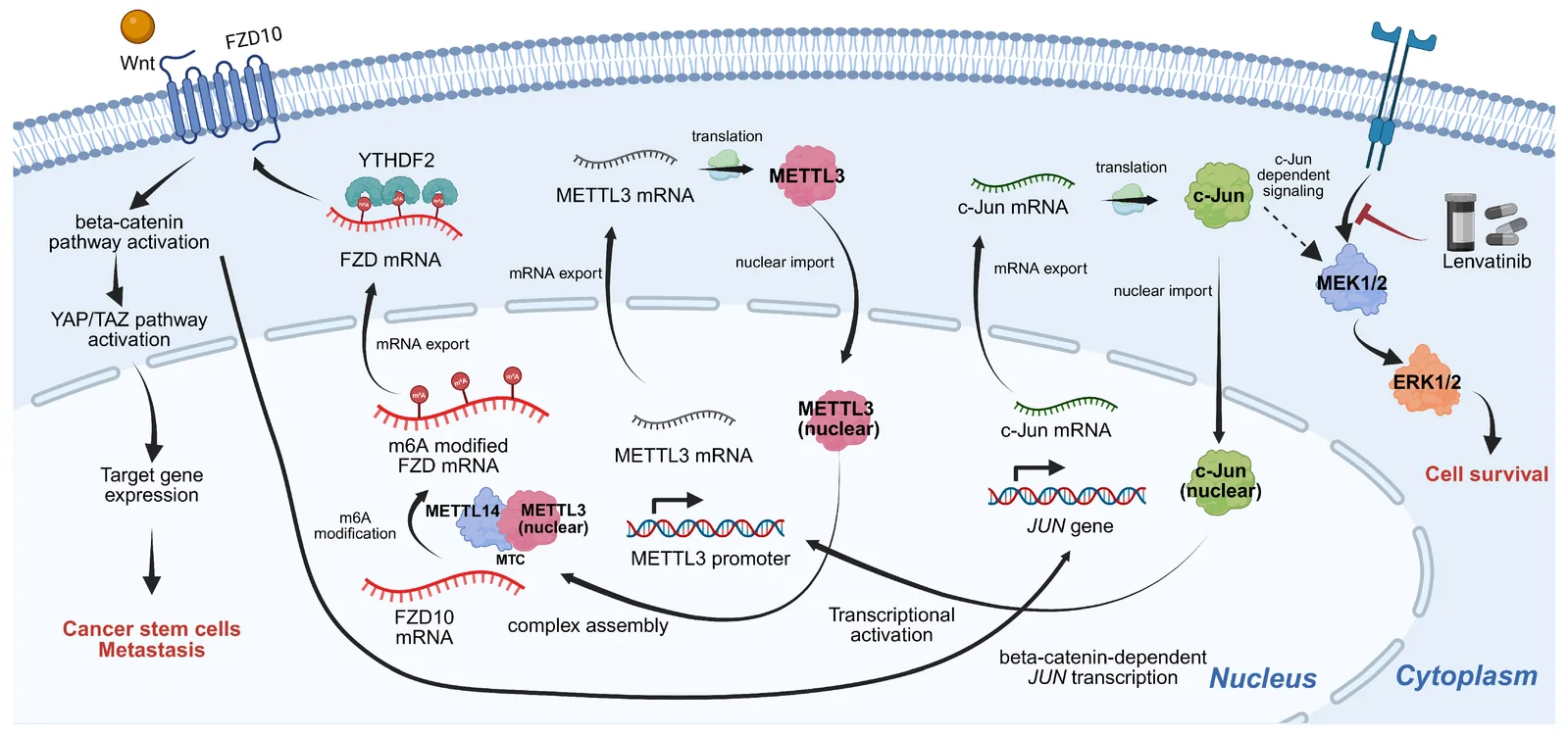
**METTL3-mediated m^6^A modification of FZD10 mRNA activates Wnt/β-catenin and YAP/TAZ signaling in HCC.** The METTL3-METTL14 complex deposits m^6^A modifications onto FZD10 mRNA, which is recognized by YTHDF2, enhancing FZD10 mRNA stability and translation. Elevated FZD10, a Wnt receptor, upon binding to the Wnt ligand, activates Wnt/β-catenin signaling, leading to nuclear accumulation of β-catenin and transcriptional upregulation of JUN, which encodes c-Jun. The c-Jun and METTL3 mRNAs are exported to the cytoplasm for translation. The newly synthesized METTL3 protein is imported into the nucleus and assembles with METTL14 into the methyltransferase complex (MTC). Newly synthesized c-Jun also enters the nucleus, where it activates METTL3 transcription, completing a positive feedback loop. The dashed arrow from c-Jun to MEK1/2 indicates pathway-level regulation rather than direct physical interaction or protein translocation. Solid arrows denote the direction of molecular processes or regulatory interactions, dashed arrows represent indirect or pathway-level regulatory relationships, and blunt-ended lines indicate inhibitory effects. Enhanced Wnt/β-catenin signaling further promotes YAP/TAZ activation, collectively driving cancer stem cell properties, metastasis, and lenvatinib resistance. In parallel, lenvatinib suppresses RTK-mediated MEK–ERK activation; however, FZD10-driven Wnt/β-catenin/c-Jun signaling sustains MEK activation independently of RTK, thereby conferring lenvatinib resistance and promoting cell survival. Created in Biorender. Hyung Seok Kim. (2026) https://BioRender.com/7ytdt7u (accessed on 30 June 2026).

METTL3 promotes Wnt/β-catenin signaling in HCC through m^6^A-dependent upregulation of the Frizzled receptor FZD10, a Wnt receptor [3,68]. Hyperactivation of the Wnt/β-catenin signaling axis occurs in approximately 20–40% of HCCs and contributes to tumor stemness, proliferation, and drug resistance [69]. Wang et al. showed that m^6^A modification of FZD10 mRNA enhances its stability and translation, increasing Wnt pathway activation (Figure 5) [68]. Enhanced Wnt signaling promotes cancer stem cell properties, lenvatinib resistance, and YAP activation in HCC [1,10]. These findings indicate that METTL3 coordinates Wnt/β-catenin and Hippo/YAP signaling through interconnected oncogenic mechanisms in HCC [8]. METTL3 upregulation is also associated with lenvatinib resistance in HCC and has been proposed as a biomarker for predicting therapeutic response [36].

#### 3.2.4. Hippo/YAP Signaling

METTL3-mediated m^6^A modification modulates the activity of the Hippo/YAP pathway, a pathway that regulates tumor growth and liver regeneration [8,70]. Transcriptomic analysis following METTL3 knockdown revealed significant enrichment of the Hippo pathway, while m^6^A profiling revealed that YAP1 mRNA is a direct target of METTL3 [70]. In addition to directly modifying YAP1 mRNA, METTL3 also activates Hippo/YAP signaling by regulating MOB1A mRNA. METTL3 promotes YTHDF2-mediated decay of MOB1A, a core component of the Hippo kinase cascade, reducing inhibitory YAP phosphorylation and promoting its nuclear accumulation to drive pro-tumorigenic transcriptional programs associated with hepatocarcinogenesis [8,70]. Loss of MOB1A disrupts canonical Hippo inhibitory signaling and promotes proliferation, survival, and stemness-associated phenotypes in aggressive HCC [70]. Activated YAP also cooperates with the Wnt/β-catenin pathway to further promote HCC progression. The crosstalk supports cancer stem cell properties and therapeutic resistance [3,10].

#### 3.2.5. EMT-Associated Pathways

Elevated METTL3 expression is associated with an enhanced epithelial–mesenchymal transition (EMT) in HCC, promoting invasive and migratory characteristics that facilitate metastatic dissemination [71,72,73]. METTL3 mediates this effect through m^6^A-dependent post-transcriptional regulation of key EMT-promoting transcription factors and signaling mediators. For example, METTL3-mediated m^6^A modification stabilizes IQGAP3 mRNA through IGF2BP2-dependent recognition, activating TGF-β/SMAD signaling and inducing EMT, which promotes HCC cell migration, invasion, and metastasis in vitro and in vivo [71]. By increasing the stability and translational efficiency of these pro-metastatic transcripts, METTL3 promotes cellular invasion, extracellular matrix remodeling, and metastatic progression [74,75]. In gastric cancer, METTL3-mediated stabilization of transcripts such as ZMYM1 facilitates EMT, suggesting a conserved mechanism across cancer types [32,76]. In cholangiocarcinoma, METTL3-dependent modulation of alternative pathways, including YTHDF2-mediated degradation of IFIT2 mRNA, also promotes tumor progression [77]. Together, these findings suggest that METTL3 promotes EMT and metastatic progression across hepatic and gastrointestinal malignancies, although the downstream effectors differ among tumor types.

#### 3.2.6. Notch Signaling

The Notch signaling pathway is a highly conserved intercellular communication cascade that regulates essential cellular processes through interactions between neighboring cells. Abnormal activation of Notch signaling has been widely implicated in HCC, where it promotes tumor initiation and progression by enhancing cell proliferation, metastasis, stem cell-like properties, and resistance to anticancer therapies [78]. In contrast, DLL3 has been reported to negatively regulate HCC cell growth by suppressing Notch signaling, although its expression is frequently silenced through promoter methylation in human HCC [79]. Consistent with the oncogenic role of Notch signaling, Wu et al. reported that high METTL3 expression in HCC tissues positively correlated with activation of the Wnt, cell cycle, and Notch signaling pathways [40]. Downstream, the YTHDF1–NOTCH1 axis directly contributes to therapeutic resistance in HCC. YTHDF1 binds to m^6^A-modified NOTCH1 mRNA, enhancing its stability and translational efficiency, which increases tumor-initiating capacity and self-renewal. Depletion of YTHDF1 increases the sensitivity of HCC cells to lenvatinib and sorafenib, whereas enforced expression of NOTCH1 promotes resistance to these therapies [80]. Notch1 activation also promotes EMT in HCC, facilitating stem cell-like properties, migration, invasion, and chemoresistance. These observations highlight the crosstalk between Notch signaling and other oncogenic pathways in HCC [81].

#### 3.2.7. Hedgehog Signaling

The Hedgehog signaling pathway is aberrantly activated in a subset of HCCs and contributes to cancer stem cell maintenance, epithelial–mesenchymal transition, and therapeutic resistance [74,82]. However, the direct contribution of METTL3-mediated m^6^A regulation to Hedgehog activation in HCC remains incompletely defined. Studies in other cancer models have shown that METTL3 can regulate Hedgehog pathway activity through m^6^A-dependent post-transcriptional mechanisms. In the Sonic Hedgehog subgroup of medulloblastoma, METTL3-mediated m^6^A modification of PTCH1 and GLI2 transcripts enhances their RNA stability and translation, activating Sonic Hedgehog signaling and promoting tumor progression [83]. In HCC, hypoxia-driven ROS-mediated non-canonical Hedgehog signaling has been implicated in EMT and may contribute to tumor progression and therapeutic resistance [72,84]. Whether a similar METTL3-dependent regulatory mechanism contributes to Hedgehog activation in HCC remains to be established.

### 3.3. Cellular Reprogramming in HCC

In addition to regulating oncogenic signaling pathways, METTL3 also influences several cellular processes that contribute to HCC progression. METTL3-mediated m^6^A modification regulates cell cycle progression, glycolytic reprogramming, autophagy, and immune evasion, enabling HCC cells to sustain proliferation under metabolic and therapeutic stress. METTL3 promotes G1/S cell cycle progression by enhancing the stability and translation of cell cycle-related mRNAs [49], while also increasing glycolytic activity through the regulation of enzymes and metabolic factors involved in aerobic glycolysis, providing the energy and biosynthetic intermediates required for continuous proliferation [28]. The increased metabolic demand of this proliferative state is offset by METTL3-dependent regulation of autophagy, which preserves cellular homeostasis by recycling intracellular components during nutrient deprivation, hypoxia, and treatment-induced stress. In HCC, persistent activation of autophagy has also been linked to improved tumor cell survival and reduced sensitivity to therapies such as sorafenib [47,85]. METTL3 also influences TIME by regulating immune checkpoint molecules and other immunomodulatory factors, suppressing antitumor immune responses, and facilitating immune escape [25,86]. Collectively, these mechanisms sustain tumor growth and reduce therapeutic sensitivity in HCC.

### 3.4. Tumor Immune Microenvironment

#### 3.4.1. Cytokine Regulation

METTL3 regulates cytokine signaling primarily through the SOCS/JAK/STAT axis. METTL3 forms a positive feedback loop with STAT3, a transcription factor activated downstream of cytokine signaling, while SOCS2 and SOCS3 function as negative regulators of this pathway by limiting cellular sensitivity to cytokine-driven growth signals [15,25,87,88]. Sustained STAT3 activity promotes transcriptional programs associated with tumor cell survival, metabolic adaptation, and oncogenic cytokine signaling [8]. METTL3-mediated m^6^A modification facilitates YTHDF2-dependent decay of SOCS2 mRNA in HCC [25,33], impairing a negative regulatory mechanism that normally constrains JAK/STAT pathway activation and sustaining STAT3-dependent transcription [15]. In HCC, METTL3 overexpression promotes YTHDF2-dependent degradation of SOCS2 mRNA, reducing SOCS2 expression and enhancing HCC cell proliferation and migration through aberrant STAT3 signaling [8,25,89].

#### 3.4.2. T-Cell Dysfunction

METTL3 contributes to T-cell dysfunction by remodeling the TME [90,91]. Activation of oncogenic pathways, such as the JAK/STAT signaling, disrupts normal immune surveillance [88,91]. The resulting metabolic and signaling changes promote an immunosuppressive microenvironment that limits antitumor immune responses in HCC. One mechanism involves METTL3-dependent m^6^A modification of SREBP cleavage-activating protein (SCAP), enhancing cholesterol biosynthesis, and impairing the anti-tumor activity of CD8+ T cells, contributing to resistance to immune checkpoint blockade [9,91]. METTL3 has been implicated in HCC immune evasion through the SMPDL3A/LRPPRC axis. METTL3-mediated m^6^A modification stabilizes SMPDL3A mRNA, thereby increasing LRPPRC expression and promoting HCC-associated immune escape [41]. In a separate HCC study, LRPPRC was shown to interact with PD-L1 mRNA and enhance its m^6^A-dependent stability; accordingly, LRPPRC depletion reduced PD-L1 mRNA and protein expression and enhanced antitumor immunity [92]. These findings suggest a possible indirect link between METTL3 and PD-L1 regulation; however, the cited studies do not establish direct METTL3-mediated m^6^A modification of PD-L1 mRNA in HCC. Taken together, the studies discussed above support a role for METTL3 in shaping the immune microenvironment of HCC.

Collectively, METTL3 regulates diverse coding and non-coding RNA targets, oncogenic signaling pathways, metabolic programs, and immune responses in HCC, as summarized in Table 1.

## 4. Therapeutic Strategies Targeting METTL3 in HCC

METTL3 overexpression across multiple cancer types and its strong correlation with poor prognosis make it an attractive therapeutic target [4,15]. Moreover, METTL3-mediated m^6^A modifications have been implicated in drug resistance. However, therapeutic strategies must account for METTL3’s context-dependent roles, as its silencing may paradoxically promote sorafenib resistance through autophagy-related pathways [8].

### 4.1. Small-Molecule and Peptide-Based Inhibition of METTL3

HCC treatment has been substantially advanced by the introduction of immune checkpoint inhibitors (ICIs), which target key regulatory pathways such as CTLA-4 and PD-1/PD-L1. These therapies have transformed treatment paradigms and demonstrated clinical efficacy in HCC [91]. However, despite these successes, limitations such as variable response and acquired resistance have driven the search for complementary therapeutic strategies. Targeting epitranscriptomic regulators such as METTL3 represents a potential therapeutic strategy. The initial discovery of METTL3 inhibitors stemmed from high-throughput screening of adenosine analogues, designed to bind the SAM binding site and inhibit methyltransferase activity [96]. Based on the crystal structure of the METTL3 catalytic pocket, recent drug development efforts have focused on SAM-competitive inhibitors that specifically target the MTD of METTL3. By mimicking the SAM cofactor, these inhibitors disrupt the catalytic activity of the METTL3–METTL14 complex enhancing selectivity and minimizing off-target interactions with other methyltransferases. Despite ongoing challenges in pharmacokinetic optimization, this rational design strategy represents a significant step toward the precise epitranscriptomic modulation in METTL3-driven HCC [36].

Although preclinical data are promising, the clinical translation of METTL3-targeted therapies, such as STC-15, remains in its early stages, with no established HCC-specific clinical efficacy data to date [13,36,95]. In Phase I (NCT05584111), STC-15 achieved target engagement and showed preliminary antitumor activity in 42 patients with advanced malignancies. The ongoing Phase 1b/2 trial (NCT06975293), evaluating STC-15 in combination with the anti-PD-1 antibody toripalimab, has an estimated enrollment of 107 participants [91,97,98]. However, HCC-specific clinical efficacy has not been established, and these findings cannot yet be directly extrapolated to HCC.

Several selective METTL3 inhibitors, including STC-15, UZH1a, and STM2457, have been developed, with some progressing into early-phase clinical trials although translating these preclinical findings into clinical practice remains challenging [6,36]. Most evidence is derived from preclinical studies, and clinical validation of safety, efficacy, and delivery strategies remains necessary [6]. Alongside small-molecule inhibitors, peptide-based strategies are being developed to disrupt METTL3 protein–protein interactions, potentially reducing immunogenicity and limiting acquired resistance [22,32,99]. Among these inhibitors, STC-15 has demonstrated both antitumor and immunomodulatory activities [100,101]. Preclinical studies indicate that STC-15 activates innate immune signaling and enhances the efficacy of immune checkpoint inhibitors, including anti-CTLA-4 and anti-PD-1/PD-L1 antibodies [91,100,101,102]. This approach may help overcome the limitations of monotherapy and improve therapeutic outcomes in HCC, supporting a potential role for METTL3 inhibition in combination immunotherapy strategies [6,9].

Similarly, the small-molecule inhibitor STM2457 has demonstrated antitumor activity and immunomodulatory effects in preclinical models. By binding to the catalytic domain of METTL3, STM2457 selectively inhibits its methyltransferase activity, reducing global m^6^A methylation. Consequently, STM2457 suppresses cell viability [65], proliferation (as indicated by decreased EdU-positive cells) [41], and invasion, while promoting apoptosis in vitro and in vivo models [26,36]. Jin et al. reported that STM2457 enhanced HCC cell sensitivity to oxaliplatin and demonstrated that the METTL3/TRIM21/G6PD axis contributes to oxaliplatin resistance, supporting METTL3 inhibition as a potential strategy for overcoming chemoresistance [65]. STM2457 has also been reported to reverse lenvatinib resistance, collectively supporting METTL3 inhibition as a strategy to overcome drug resistance in HCC [26,36]. Furthermore, combining STM2457 with sorafenib improved therapeutic responsiveness by disrupting METTL3-driven activation of the serine synthesis pathway and impairing the metabolic and antioxidant adaptation of resistant HCC cells [67]. Collectively, these findings support METTL3 inhibition, either alone or in combination with existing therapies, as a promising strategy for overcoming drug resistance in HCC.

### 4.2. CRISPR-Cas9-Based Strategies

Studies using METTL3 knock-in, CRISPR-Cas9 knockout, and knockdown approaches have established METTL3 as a critical regulator of tumor cell survival, particularly in non-alcoholic fatty liver disease (NAFLD)-associated HCC [9]. These studies consistently demonstrate that METTL3 ablation attenuates tumor cell proliferation, survival, and metastatic potential [4,9,24]. Although METTL3 ablation suppresses tumor progression in most models, enhanced hepatocarcinogenesis has been reported in specific contexts, indicating context-dependent roles of METTL3 [24]. This functional divergence suggests that METTL3 can assume protective roles under specific conditions, as evidenced by its modulation of adaptive autophagy pathways. METTL3 silencing may activate protective autophagy that promotes sorafenib resistance [8], potentially limiting the efficacy of METTL3-targeted therapies. Collectively, these findings indicate that the therapeutic effects of METTL3 inhibition are context dependent, necessitating consideration of tumor-specific dependencies and resistance mechanisms in therapeutic development.

### 4.3. Combination Immunotherapy in HCC

METTL3 modulation may enhance the efficacy of immune checkpoint blockade. Targeting METTL3 has been shown to synergize with anti-PD-1 therapy, leading to robust tumor growth inhibition, including >90% reductions in tumor volume and weight compared with the control, by reinvigorating CD8+ T-cell function and alleviating the immunosuppressive microenvironment [9,91]. Characterizing the regulatory crosstalk between METTL3 and immune checkpoints such as PD-L1 offers a compelling rationale for incorporating epitranscriptomic-targeted therapies into combination regimens to counteract immune evasion in HCC [5]. Therapeutic strategies targeting the METTL3–METTL14 complex are being developed to disrupt m^6^A-dependent oncogenic signaling. In addition to conventional catalytic inhibition, emerging strategies such as PROTAC-mediated degradation, exemplified by WD6305, aim to eliminate the methyltransferase machinery through targeted protein degradation. This approach may offer advantages over conventional enzymatic inhibition by achieving more complete suppression of METTL3 activity [103,104].

### 4.4. Delivery Systems Targeting Liver Tumors

Efficient drug delivery remains a critical bottleneck for the clinical translation of METTL3-targeted therapies in HCC [6]. The therapeutic utility of small-molecule METTL3 inhibitors is often limited by poor water solubility, low bioavailability, and potential systemic off-target toxicity [48]. To overcome these challenges, future research must focus on optimizing advanced delivery systems, including nanoparticle-based carriers, to achieve tumor-selective delivery within the hepatic TME while minimizing systemic toxicity [6,48]. Approaches like the use of ligand-conjugated or stimulus-responsive nanoparticles have demonstrated potential to enhance drug stability, improve tumor penetration, and facilitate controlled release within the TME [48]. Furthermore, nanoparticle-based delivery of genetic agents such as siMETTL3 has demonstrated robust antitumor immune responses and improved efficacy in combination with immunotherapy in preclinical models [9,48].

Taken together, current pharmacological, genetic, combination, and delivery-based strategies targeting METTL3 in HCC are summarized in Table 2.

## 5. Current Challenges and Future Perspectives

Despite substantial evidence supporting METTL3 as an important regulator of HCC progression, several challenges remain before METTL3-directed strategies can be translated into clinically effective therapies. Key issues include the context-dependent functions of METTL3, patient selection, biomarker development, treatment resistance and potential on-target toxicity, and the limited clinical validation of METTL3-targeted therapies.

The role of METTL3 may differ across the cellular and molecular contexts and stages of hepatocarcinogenesis. Experimental evidence suggests that METTL3 may exert tumor-suppressive effects during early hepatocarcinogenesis, in contrast to its previously reported oncogenic role in established HCC [24]. Therefore, the effects of METTL3 inhibition may depend on the stage of tumor development. In addition, METTL3 activity may differ between malignant hepatocytes and immune or stromal cells within the tumor microenvironment. Thus, METTL3 expression measured in bulk tumor tissue may not fully reflect METTL3 dependency in malignant cells [4,5,91].

Reliable biomarkers are also needed for patient selection, prognosis, and therapeutic monitoring [13,107]. Clinical evidence supports a role for METTL3 in HCC progression, prognosis, and therapeutic resistance. METTL3 was significantly upregulated in 50 paired HCC and normal liver tissues. In the same study, an analysis of 360 HCC patients using TCGA and cBioPortal data identified METTL3 expression as an independent prognostic factor for overall survival (multivariate HR = 2.046, 95% CI: 1.303–3.212, *p* = 0.002) and recurrence-free survival [20]. In an independent analysis of 10 paired HCC and adjacent non-tumor tissues, METTL3 and STAT3 protein levels were significantly elevated in HCC tissues, particularly in metastatic lesions. Analysis of tissue microarrays comprising 122 clinical specimens further demonstrated a strong positive correlation between METTL3 and STAT3 protein expression (r = 0.656, *p* < 0.001), while functional studies showed that the METTL3–STAT3–WTAP positive-feedback loop promoted HCC metastasis [15]. In a cohort of 74 HCC patients receiving adjuvant lenvatinib after radical treatment, high METTL3 expression was associated with poorer overall survival (37 patients each in the high- and low-expression groups). METTL3 expression was also higher in lenvatinib-resistant than in lenvatinib-sensitive HCC tissues. In preclinical models, METTL3 inhibition with STM2457 enhanced lenvatinib sensitivity by suppressing EGFR translation [26]. These findings support the prognostic and therapeutic potential of METTL3, although its predictive value for METTL3-targeted therapy remains unclear. Circulating m^6^A-related markers may also have monitoring potential but require prospective validation [74,107].

Therapeutic resistance and toxicity remain additional concerns. METTL3-mediated m^6^A regulation has been implicated in resistance to therapies such as sorafenib, highlighting the need to understand acquired resistance and develop rational combination strategies [64,85,108]. Meanwhile, because METTL3 also regulates physiological processes such as hepatic lipid metabolism, systemic inhibition may affect normal liver function. Poor drug bioavailability and limited target specificity may further restrict clinical translation, emphasizing the need for an adequate therapeutic window and improved tumor selectivity [13,108].

Several therapeutic strategies targeting METTL3, including selective small-molecule inhibitors and approaches that disrupt MTC function, have demonstrated promising preclinical activity. However, improving target specificity, establishing long-term safety, and achieving tumor-selective delivery remain important priorities for clinical development. Rational combination strategies with established targeted therapies or immunotherapies may also improve therapeutic efficacy while limiting systemic effects [6,109].

**Figure 6:**
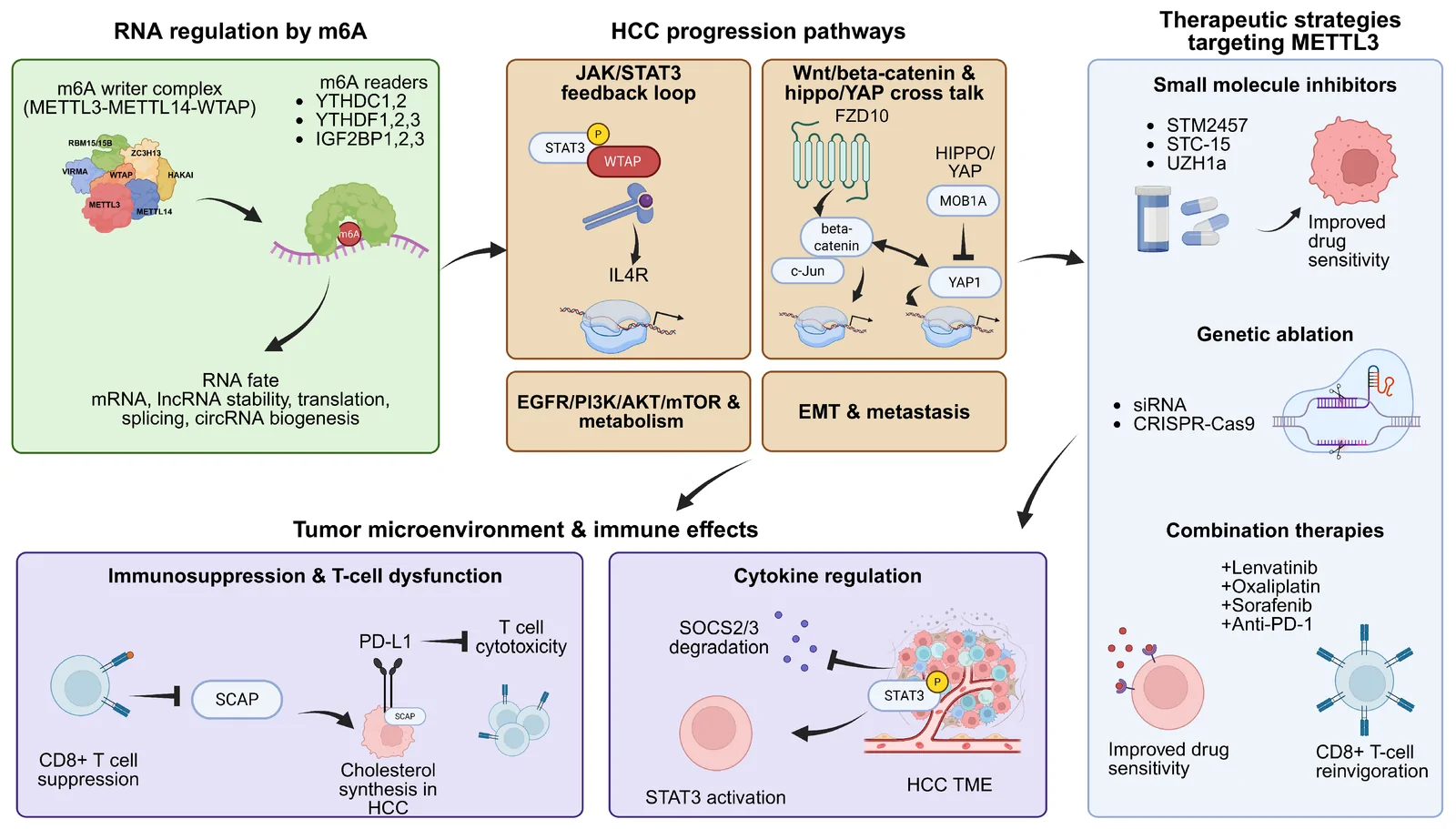
**Comprehensive schematic of METTL3-mediated epitranscriptomic regulation and therapeutic targeting in hepatocellular carcinoma (HCC).** The diagram illustrates the interconnected roles of the METTL3–METTL14 m^6^A writer complex across four key domains: (1) RNA regulation, where m^6^A deposition dictates RNA fate via reader proteins; (2) HCC progression pathways, where m^6^A-modified transcripts activate critical oncogenic cascades including JAK/STAT3, Wnt/β-catenin, Hippo/YAP, and EGFR signaling; (3) Tumor microenvironment and immune effects, depicting how these pathways drive metabolic reprogramming (e.g., SCAP-mediated cholesterol synthesis) and cytokine regulation to induce T-cell dysfunction and immunosuppression; and (4) Therapeutic strategies, highlighting the translation of these mechanisms into actionable interventions, including small-molecule inhibitors, genetic ablation, and combination therapies to improve drug sensitivity and reinvigorate anti-tumor immunity.Created in Biorender. Hyung Seok Kim. (2026) https://BioRender.com/jwpjlc8 (accessed on 30 June 2026).

Future studies should integrate multi-omics, single-cell, and spatial approaches with improved preclinical models to delineate the molecular determinants governing METTL3 activity across distinct cellular populations within the HCC microenvironment, identify predictive biomarkers, and clarify resistance mechanisms [4,9,74,108]. From a therapeutic perspective, more selective METTL3 inhibitors and targeted delivery strategies could be explored in combination with immunotherapy, radiotherapy, chemotherapy, or established targeted therapies such as sorafenib [109]. Prospective HCC trials with appropriate biomarker stratification, pharmacodynamic assessment, and long-term safety evaluation are needed to determine the clinical benefit of METTL3-targeted therapy [13,36]. The overarching connections between METTL3-mediated RNA regulation, oncogenic signaling, immune microenvironment remodeling, and targeted therapeutic strategies discussed in this review are summarized in Figure 6.

## 6. Conclusions

METTL3 has emerged as a central regulator of m^6^A RNA methylation, playing a pivotal role in the development and progression of HCC. Through post-transcriptional regulation of both coding and non-coding RNAs, METTL3 orchestrates multiple biological processes, including oncogenic signaling, RNA metabolism, metabolic reprogramming, immune evasion, and therapeutic response. Collectively, these diverse functions position METTL3 at the nexus of the epitranscriptomic networks that drive HCC progression, supporting its value as both a prognostic biomarker and a therapeutic target.

Accumulating evidence supports METTL3 as a promising therapeutic target, with selective small-molecule inhibitors and approaches targeting the MTC demonstrating encouraging preclinical activity. However, the context-dependent functions of METTL3, together with challenges related to patient selection, target specificity, therapeutic resistance, and long-term safety, remain important considerations for clinical translation. Although METTL3 inhibitors have entered early clinical evaluation, their clinical efficacy in HCC remains to be established.

Overall, further integration of mechanistic, epitranscriptomic, and clinical studies will be required to define the therapeutic potential of METTL3 in HCC. Such efforts may support the development of more precise METTL3-targeted strategies and identify patients most likely to benefit from these approaches.

## Figures and Tables

**Table 1:** Major METTL3-regulated targets, m^6^A-dependent mechanisms, and biological consequences in HCC.

Biological Process/Pathway	Molecular Target(s)	m^6^A-Dependent Molecular Mechanism	Biological Consequence	References
JAK2/STAT3 signaling	IL4R mRNA, IGF2BP3, WTAP	IGF2BP3 stabilizes m^6^A-modified IL4R mRNA, activating JAK2/STAT3 signaling. STAT3-induced WTAP enhances METTL3 nuclear retention, establishing a positive feedback loop.	Promotes proliferation, migration, invasion, metastasis, and sustained JAK2/STAT3 activation.	[17]
Cytokine signaling regulation	SOCS2, SOCS3 mRNAs	METTL3 promotes YTHDF2-dependent degradation of SOCS2 and reduces SOCS3 stability, relieving negative regulation of JAK/STAT signaling.	Enhances tumor growth, liver cancer stemness, and tumorigenesis.	[25,87]
Pentose phosphate pathway (PPP)	TRIM21/G6PD axis	METTL3-mediated m^6^A promotes YTHDF2-dependent degradation of TRIM21, stabilizing G6PD and activating the PPP.	Increases NADPH production, antioxidant capacity, cell survival, and oxaliplatin resistance.	[65]
Serine synthesis pathway (SSP)	PHGDH, PSAT1, PSPH, PCK2, NRF2 transcripts	METTL3 recruits IGF2BP3 to stabilize SSP transcripts, while lactylated IGF2BP3 reinforces serine metabolism and antioxidant signaling through PCK2 and NRF2.	Enhances serine biosynthesis, redox homeostasis, therapeutic adaptation, and resistance to sorafenib and lenvatinib.	[67,93]
Sorafenib response and hypoxic adaptation	FOXO3 mRNA	METTL3-mediated m^6^A modification of FOXO3 mRNA enhances transcript stability through YTHDF1-dependent recognition.	Maintains FOXO3 expression and suppresses hypoxia-associated sorafenib resistance by regulating angiogenesis- and autophagy-related responses.	[85]
Wnt/β-catenin signaling	FZD10, LIMA1 mRNAs	METTL3-mediated m^6^A modification enhances the stability and expression of FZD10 and LIMA1 transcripts, promoting activation of Wnt/β-catenin signaling.	Activates Wnt/β-catenin signaling, enhancing cancer stem cell properties, proliferation, migration, invasion, and resistance to lenvatinib.	[1,10,68]
Hippo/YAP signaling	YAP1 mRNA, LIMA1, lnc-CTHCC	METTL3-mediated m^6^A modification enhances YAP1 translation and regulates YAP-associated transcripts. In addition, METTL3-dependent stabilization of lnc-CTHCC promotes YAP1 transcriptional activation, while LIMA1-mediated regulation contributes to Hippo pathway activation.	Enhances YAP activity, promoting vasculogenic mimicry, cancer stemness, proliferation, invasion, metastasis, and tumor progression.	[57,70]
EGFR signaling	EGFR mRNA	METTL3-mediated m^6^A increases EGFR expression and sustains downstream signaling.	Supports tumor survival and contributes to lenvatinib resistance.	[26]
mRNA stability regulation	USP7 mRNA	METTL3-mediated m^6^A increases USP7 expression.	Promotes proliferation, migration, and invasion.	[94]
Cell-cycle progression and DNA replication	MCM10 mRNA	METTL3 stabilizes MCM10 mRNA through IGF2BP3-dependent recognition.	Enhances DNA replication, proliferation, migration, invasion, and tumor growth.	[22]
EMT and metastatic progression	IQGAP3 mRNA	METTL3-mediated m^6^A stabilizes IQGAP3 through IGF2BP2, activating TGF-β/SMAD signaling.	Promotes EMT, invasion, migration, and lung metastasis.	[71]
Immune microenvironment remodeling	SCAP	METTL3 enhances SCAP translation, increasing cholesterol biosynthesis and creating an immunosuppressive TME.	Suppresses CD8^+^ T-cell activity, promotes immune escape, and reduces PD-1 blockade efficacy.	[9]
Polycomb-mediated epigenetic regulation	BMI1, RNF2	METTL3-mediated m^6^A enhances IGF2BP1/2/3-dependent stabilization of BMI1 and RNF2 transcripts.	Promotes proliferation, invasion, tumor growth, and HCC progression.	[95]
miRNA maturation	pri-miR-93	METTL3-mediated m^6^A promotes maturation of pri-miR-93 into miR-93-5p, suppressing CDKN1A.	Accelerates cell-cycle progression, proliferation, and apoptosis resistance.	[46]
lncRNA regulation	GBAP1, LINC00958, lnc-CTHCC, MAAS	METTL3-mediated m^6^A regulates lncRNA stability or expression, thereby modulating downstream miRNA, RNA-binding protein, and signaling networks.	Promotes proliferation, invasion, lipid metabolic reprogramming, YAP activation, macrophage-mediated progression, and metastasis.	[55,56,57,58]
circRNA regulation	circ-CCT3, circ-ARL3, circHPS5	METTL3-mediated m^6^A regulates circRNA biogenesis, stability, and YTHDC1-dependent nuclear export.	Promotes cancer stemness, EMT, proliferation, invasion, and metastasis.	[53,54,61]

Note: Unless otherwise indicated, all entries are based on studies conducted in HCC models.

**Table 2:** Therapeutic strategies targeting METTL3 in HCC: mechanisms, preclinical evidence, and translational status.

Therapeutic Strategy	Representative Agent	Therapeutic Mechanism	HCC Evidence	Translational Status	References
Small-molecule METTL3 catalytic inhibition	STM2457	Suppresses METTL3-dependent m^6^A methylation and oncogenic signaling	Inhibits proliferation, invasion, tumor growth; induces apoptosis; remodels transcriptome	Preclinical	[4]
Combination with targeted therapy	STM2457 + sorafenib/lenvatinib	STM2457 inhibits METTL3-dependent serine synthesis pathway activation or EGFR upregulation, thereby enhancing sensitivity to sorafenib or lenvatinib, respectively.	Improves sorafenib response and makes lenvatinib effective in resistant HCC cells (such as Huh7-LR and MHCC97H) and multiple mouse models (subcutaneous, orthotopic, and hydrodynamic models).	Preclinical	[26,67]
Combination with chemotherapy	STM2457 + oxaliplatin	Suppresses the METTL3-TRIM21/G6PD pathway, increases oxidative stress and enhances DNA damage.	Restores oxaliplatin sensitivity in organoids and orthotopic models	Preclinical	[65]
Combination immunotherapy	STM2457, siMETTL3 or sgMETTL3 + anti-PD-1	Restores CD8^+^ T-cell activity through inhibition of SCAP/cholesterol metabolic reprogramming.	Strong efficacy in orthotopic NAFLD-HCC models	Preclinical	[9]
Genetic suppression	siRNA, shRNA, CRISPR	Direct depletion of METTL3	Target validation across numerous HCC models	Experimental	[4,9,24]
Peptide-mediated inhibition	METTL3 O-GlcNAcylation peptides	Destabilizes METTL3 and disrupts WTAP interaction	Suppresses tumor progression	Early preclinical	[22]
Emerging METTL3-targeted inhibitors	STC-15, UZH1a, WD6305	Direct inhibition of METTL3 catalytic activity by STC-15, and UZH1a or targeted PROTAC-mediated degradation by WD6305, resulting in suppression of m^6^A modification	HCC-specific clinical efficacy has not yet been established. Preclinical activity has been reported for UZH1a and WD6305, primarily in non-HCC cancer models.	STC-15: Phase I (NCT05584111) Phase 1b/2 (NCT06975293). UZH1a and WD6305: preclinical stage.	[97,98,100,101,104,105,106]

Note: Unless otherwise indicated, all entries are based on studies conducted in HCC models.

## Data Availability

No new data were created or analyzed in this review article. Data sharing is not applicable to this article. Figure 1, Figure 2, Figure 3, Figure 4, Figure 5 and Figure 6 were created using BioRender (http://www.BioRender.com) (accessed on 30 June 2026).

## Abbreviations

ACSL5	acyl-CoA synthetase long-chain family member 5
AKT	AKT serine/threonine kinase
ALKBH5	AlkB homolog 5, RNA demethylase
BAMBI	BMP and activin membrane-bound inhibitor
BCLC	Barcelona Clinic Liver Cancer Group
BMI1	BMI1 proto-oncogene, polycomb ring finger
BMPR1A	bone morphogenetic protein receptor type 1A
CDKN1A	cyclin-dependent kinase inhibitor 1A
circRNA	circular RNA
CPSF5/CPSF6	cleavage and polyadenylation specificity factor subunit 5/6
CRISPR-Cas9	clustered regularly interspaced short palindromic repeats/CRISPR-associated protein 9
CTLA-4	cytotoxic T-lymphocyte-associated protein 4
DLL3	delta-like canonical Notch ligand 3
EdU	5-ethynyl-2′-deoxyuridine
EGFR	epidermal growth factor receptor
EMT	epithelial–mesenchymal transition
FEN1	flap structure-specific endonuclease 1
FLT1	fms-related receptor tyrosine kinase 1
FOXO3	forkhead box O3
FOXP4	forkhead box P4
FTO	fat mass and obesity-associated protein
FZD10	frizzled class receptor 10
G6PD	glucose-6-phosphate dehydrogenase
GBAP1	glucosylceramidase beta pseudogene 1
GLI2	GLI family zinc finger 2
HAKAI (CBLL1)	Casitas B-lineage lymphoma-transforming sequence-like protein 1
HBx	hepatitis B virus X protein
HBXIP	hepatitis B X-interacting protein
HCC	hepatocellular carcinoma
HDGF	hepatoma-derived growth factor
HMGA2	high mobility group AT-hook 2
hnRNPK	heterogeneous nuclear ribonucleoprotein K
ICI	immune checkpoint inhibitor
IFIT2	interferon-induced protein with tetratricopeptide repeats 2
IGF2BP1/2/3	insulin-like growth factor 2 mRNA-binding protein 1/2/3
IL4R	interleukin 4 receptor
IQGAP3	IQ motif containing GTPase activating protein 3
JAK	Janus kinase
KLF2	Krüppel-like factor 2
LIMA1	LIM domain and actin binding 1
LINC00958	long intergenic non-protein coding RNA 958
lncRNA	long non-coding RNA
m^6^A	N^6^-methyladenosine
MAAS (MAPKAPK5-AS1)	MAPK-activated protein kinase 5 antisense RNA 1
MAPK	mitogen-activated protein kinase
MCM10	minichromosome maintenance 10 replication initiation factor
METTL3	methyltransferase-like 3
METTL14	methyltransferase-like 14
miRNA	microRNA
MOB1A	MOB kinase activator 1A
MTC	m^6^A methyltransferase complex
MTD	methyltransferase domain
mTOR	mechanistic target of rapamycin
MYC	MYC proto-oncogene, bHLH transcription factor
NADPH	nicotinamide adenine dinucleotide phosphate
NAFLD	non-alcoholic fatty liver disease
NOTCH1	notch receptor 1
NRF2	nuclear factor erythroid 2-related factor 2
NSCLC	non-small-cell lung cancer
O-GlcNAc	O-linked β-N-acetylglucosamine
PCK2	phosphoenolpyruvate carboxykinase 2
PD-1	programmed cell death protein 1
PD-L1	programmed death-ligand 1
PHGDH	phosphoglycerate dehydrogenase
PI3K	phosphoinositide 3-kinase
PPARGC1A	peroxisome proliferator-activated receptor gamma coactivator 1-alpha
PPP	pentose phosphate pathway
pri-miRNA	primary microRNA
PROTAC	proteolysis-targeting chimera
PSAT1	phosphoserine aminotransferase 1
PSPH	phosphoserine phosphatase
PTCH1	patched 1
PTEN	phosphatase and tensin homolog
RBM15/15B	RNA-binding motif protein 15/15B
RICTOR	rapamycin-insensitive companion of mTOR
RNF2	ring finger protein 2
ROS	reactive oxygen species
SAH	S-adenosyl-L-homocysteine
SAM	S-adenosyl-L-methionine
SCAP	SREBP cleavage-activating protein
shRNA	short hairpin RNA
siRNA	small interfering RNA
SMAD	mothers against decapentaplegic homolog
SOCS	suppressor of cytokine signaling
SPHK2	sphingosine kinase 2
SREBP	sterol regulatory element-binding protein
SSP	serine synthesis pathway
STAT3	signal transducer and activator of transcription 3
TGF-β	transforming growth factor beta
TIME	tumor immune microenvironment
TME	tumor microenvironment
TRIM21	tripartite motif containing 21
TSC2	TSC complex subunit 2
USP7	ubiquitin-specific peptidase 7
UTR	untranslated region
VCR	variable charged region
VIRMA (KIAA1429)	Vir-like m^6^A methyltransferase-associated protein
WTAP	Wilms’ tumor 1-associating protein
YAP1	Yes-associated protein 1
YES1	YES proto-oncogene 1, Src family tyrosine kinase
YTHDC1	YTH domain-containing protein 1
YTHDF1/2	YTH N^6^-methyladenosine RNA-binding protein 1/2
ZC3H13	zinc finger CCCH-type containing 13
ZFD	zinc finger domain
ZMYM1	zinc finger MYM-type containing 1
